# New Insights into Chemical and Mineral Composition of Donkey Milk throughout Nine Months of Lactation

**DOI:** 10.3390/ani9121161

**Published:** 2019-12-17

**Authors:** Massimo Malacarne, Andrea Criscione, Piero Franceschi, Salvatore Bordonaro, Paolo Formaggioni, Donata Marletta, Andrea Summer

**Affiliations:** 1 Department of Veterinary Science, University of Parma, Via del Taglio 10, I-43126 Parma, Italy; massimo.malacarne@unipr.it (M.M.); piero.franceschi@unipr.it (P.F.); paolo.formaggioni@unipr.it (P.F.); andrea.summer@unipr.it (A.S.); 2 Department of Agriculture, Food and Environment, University of Catania, Via Valdisavoia 5, I-95131 Catania, Italy; a.criscione@unict.it

**Keywords:** Ragusano breed, donkey milk, mineral, macro and micro element, lactation

## Abstract

**Simple Summary:**

Donkey milk, because of its nutritional quality, is a more interesting product for the human diet. Moreover, due to its similarity to human milk, it is an optimal substitute for breast milk for babies. The aim of this study is to provide new insights on donkey milk production, which characterizes gross and mineral composition of Ragusano donkey breed milk. Compared to cow milk, donkey milk is characterized by a lower content of dry matter, high lactose amount, low protein, and a very low-fat content. In addition, the casein content is lower than cow’s milk. The most abundant macro element was K, followed by Ca, Na, and P. Most of the constituents are diminishing their content during lactation. In conclusion, the Ragusano breed showed a good aptitude for milk production. The composition of the milk confirmed its nutritional quality. Moreover, this research can have important positive impacts. The Ragusano breed has a long lactation and a high level of production that can ensure good income for farmers. Moreover, given its nutritional values, a larger consumption of donkey milk would be desirable and could promote the breeding of the donkey, which is an endangered species all over the world, as a profitable alternative for farmers.

**Abstract:**

Donkey milk is increasingly being proposed as a natural alternative milk for various categories of consumers, especially infants and the elderly population. However, its potential production, gross, and mineral composition have not been deeply investigated yet. Sixty-two individual milk samples were collected monthly from nine Ragusano donkeys reared in a specialized dairy farm. Milk yield as well as chemical and mineral composition, including macro and micro elements, were investigated over an entire lactation, from the second to the ninth month of milking. Milk yield averaged 1.64 kg/day, which highlights good aptitude of the Ragusano breed for the production of milk. Gross composition was characterized by low content of dry matter (8.19%), a high amount of lactose (6.07%), low protein (1.34%), and very low-fat content (0.16%). Whey proteins represented 58% of the total protein, and proteose-peptones accounted for 0.35 ± 0.07 g per 100 g. Total ash content was 0.36 g per 100 g and represented 4.40% of the dry matter. The most abundant element was K, which was followed by Ca, Na, and P. As expected, the micro elements Fe, Zn, and Cu were found in low amounts or in traces. Dry matter, fat, whey proteins. The total ash, Ca, P, Mg, and mineral ratios were significantly affected by the lactation stage.

## 1. Introduction

In the last few years, in different countries, including Italy, donkey milk has become a commercially interesting product because of the growing demand by various categories of consumers. In fact, although this milk is particularly suitable for patients suffering from cow milk’s protein allergies [1], it has a range of nutritional claims, confirmed by recent studies, especially those related to its high digestibility [2], antiviral [3], antimicrobial [4], antioxidant, and anti-inflammatory properties, as recently reviewed [5]. For these peculiar health-promoting properties, donkey milk is defined as “nutraceutical” and “pharmafood” and considered a suitable matrix to deliver a fermented beverage of new generations [6]. Despite the increasing consumption of donkey milk and the renewed interest shown by the scientific community, the research studies are still rather limited and few papers have dealt with the general characteristics of donkey milk production. Changes in the milk yield and composition throughout the lactation have been poorly investigated. In particular, few studies have described a lactation period longer than five or six months [7,8,9,10], despite that, in the specialized dairy farms, asses are milked for 9–10 months, which fully exploits their productive potential. In the panorama of the Italian donkey breeding, the Ragusano donkey is one of the most productive breeds and is employed in specialized farms for the production of milk. The Ragusano donkey was officially recognized as a breed in 1953, when, after the selection made by the “lstituto di Incremento Ippico” of Catania, the morpho-metric characteristics and the breed standard were established. The Ragusano donkey is a dark bay, with a white belly, white outline of the eyes, grey muzzle, and height at the withers between 130 and 140 cm. The average body weight of adult jennies is around 300–350 kg. Today, Ragusano is included among the native Sicilian breeds at risk of extinction. Thanks to the rediscovery of the use of donkey’s milk in the last 10 years, the Ragusano donkey has shown an increase in the number of animals (about 3500 subjects [11]). The breed is mainly addressed for the production of milk and meat as well as for recreational activities, for trekking, and for onotherapy.

The aim of this study was to investigate the detailed chemical composition of milk, including macro mineral and micro mineral elements, of the Ragusano breed reared in a specialized dairy farm, from the second to the ninth month of lactation.

## 2. Materials and Methods

All the animals involved in this research were reared under real commercial farm conditions. Therefore, no pain, suffering, distress, or lasting harm was caused to the animals involved in the present study. Milk samples used for the analyses were collected by authorized personnel during the periodic veterinary control. 

### 2.1. Animals, Diet, and Sampling

The trial was carried out in a specialized dairy farm on nine clinically healthy Ragusano jennies (2–13 parities, 40–69 days from foaling at the beginning of the trial). The age of the donkeys ranged from 6 to 16 years, the weight from 320 to 350 kg. The animals, reared indoor with external paddocks, were fed with 7 kg/day of a pelleted total mixed ration (chemical composition: crude protein 14%, fat 3%, crude fiber 15%, ash 4%) consisting of oats, barley, fava bean, wheat straw, with mineral (copper oxide 5 mg/kg, and sodium selenite mg 0.025/kg) and vitamin supplementation (vitamin A UI 15,000/kg, vitamin D3 UI 3000/kg, vitamin E 30 mg/kg). Mixed grass hay and fresh water were available ad libitum. Jennies were machine-milked twice a day at 11 a.m. and at 3 p.m. using a wheeled trolley type milking machine (adapted from the sheep and goat type) operating at 42 kPa. The foals were located together with their dams after the afternoon’s milking (3 p.m.) until 6 a.m., i.e., 5 hours before the morning milking, which is the same time interval between the two milking intervals (11 a.m.–3 p.m.). The sampling procedure used in this work is in accordance with the indications of the Italian Breeders Association to carry out functional controls in the asinine species. From the second to the ninth month of lactation, individual milk yield was recorded every month. In order to assess the chemical composition, at the morning milking (11 a.m.), individual milk samples were collected in metal-free polietilene tubes and were added with Bronopol (0.3% *v*/*v*) as a preservative. Aliquots were kept at 4 °C or frozen at −20 °C until the analysis.

### 2.2. Analytical Methods

The following parameters were determined on each milk sample: lactose and fat by means of the infrared analysis with Milko-Scan (Foss Electric, Hillerød, Denmark), pH by a potentiometer with a specific electrode (Crison Instruments, Barcelona, Spain), and somatic cells and total bacterial count by fluoro-opto-electronic and flow cytometry methods with Fossomatic 250 and BactoScan FC, respectively (Foss Electric, Hillerød, Denmark). Dry matter and ash were determined by drying at 102 °C for 7 hours and calcination in the muffle at 530 °C, respectively, and from ash solubilized in HCl (2N). The total contents of Ca, Mg, Na, K, Zn, Fe and Cu were assessed by Atomic Absorption Spectrometry (AAS) and phosphorus (P) was assessed with the colorimetric method, according to Malacarne et al. [12]. Total nitrogen (TN) on whole milk, non-casein nitrogen (NCN) on milk acid whey at pH 4.6, and non-protein nitrogen (NPN) on trichloroacetic acid (TCA) filtrated and obtained from milk after treatment with TCA (120 g/L) were determined with the Kjeldahl method. Proteose-peptone N (PPN) was measured on acid whey obtained, according to van Boeckel and Crijns [13]. From these nitrogen fractions, the following parameters were calculated: casein nitrogen (CN = TN – NCN), true protein nitrogen (TPN = TN−NPN), crude protein (TN × 6.38), casein (CN × 6.38), true protein (TPN × 6.38), and proteose-peptones (PPN × 6.38). The Kjeldahl method was performed using a DK6 Digestor and UDK126A Distiller (VELP Scientifica, Usmate, Italy), according to the Association of Official Analytical Chemists (AOAC) standards [14,15,16].

Chloride (Cl^−^) was assessed by titration with silver nitrate (AgNO_3_) using the volumetric method of Charpentier-Volhard.

All glasses and polyethylene tubes used for collection, storage, and analysis of samples were previously washed with 2N hydrochloric acid (Carlo Erba reagent, Milano, Italy) solution. All solutions were prepared using two-time distilled water (conductivity < 4.3 µS/cm).

### 2.3. Statistical Analysis

The statistical analysis was performed by Statistical Analysis Software (SAS) (SAS Inc., Cary, NC, USA). Least square means were calculated according to the following general linear model.

Y_ijk_ = µ + M_i_ + D_j_ + ε_ijk_

where Y_ijk_ = dependent variable, µ = overall mean, M_i_ = effect of class of day in milking (class 1, from 40 to 69, class 2, from 70 to 99, class 3, from 100 to 129, class 4, from 130 to 159, class 5, from 160 to 189, class 6, from 190 to 219, class 7, from 220 to 249, class 8, from 250 to 279 day in milking) (I = 1,…8), D_j_ = effect of donkey (j = 1,…9), ε_ijk_ = residual error. To evaluate the effects of the day in milking (DIM) classes, data were processed by analysis of variance for repeated measures. Pearson correlation coefficients were calculated with IBM SPSS statistics software, version 24 (Armonk, NY, USA). Somatic cell values were previously logarithmically transformed. The significant correlations were considered strong (r > 0.7), moderate (0.7 < r > 0.3), and weak (r < 0.3), according to Fantuz et al. [17].

## 3. Results

### 3.1. Milk Yield and Composition of Ragusano Donkey’s Milk

The descriptive statistics of chemical parameters, total bacterial count, and somatic cell count of donkey milk produced by Ragusano jennies are given in Table 1.

Daily milk yield was on average 1.64 kg. It must specify that this milk is the milk produced after the removal of the foal from the mother. Gross composition was characterized by a low dry matter content and a high amount of lactose. A low protein (1.34 ± 0.20 g per 100 g) and a very low-fat content (0.16 ± 0.06 g/100 g) were recorded. Whey proteins (0.78 ± 0.09 g per 100 g) represented 58% of the total protein. Proteose-peptones accounted for 0.35 ± 0.07 g per 100 g, which is about 26% of the protein fraction. Casein content showed a wide range of values and the casein to whey protein ratio value was, on average, 0.72. A notable variability was also recorded for pH values as well as total bacterial and somatic cell counts. 

The descriptive statistics of macro mineral and micro mineral content of the donkey milk produced by Ragusano jennies are given in Table 2. Total mineral content was 0.36 g per 100 g, whereas the ash percentage of the dry matter was 4.40% over the investigated period. K was the most abundant macro element (Table 2), which is followed by Ca, Na, and P. Furthermore, the mean Ca to P ratio was very close to 1 and the Na to K ratio value was 0.70. The micro elements Fe, Zn, and Cu were found in a low amount or in traces, with average content never higher than 2.5 mg/kg.

### 3.2. Effect of Lactation Stage

Table 3 shows the variation of chemical composition parameters and physico-chemical properties of Ragusano donkey milk during the lactation. Milk yield showed values of about 2 kg/day until 160 DIM, and then progressively diminished till 0.13 kg/day at 250-279 DIM. Dry matter showed a progressive lowering, with some oscillations, from the beginning (8.60 g per 100 g) to the end of lactation (7.70). The same trend was found for lactose, from 6.30 to 5.70 g per 100 g, whereas the fat content highlighted the maximum in the interval 100-219 DIM. All the nitrogen fractions were significantly affected by the lactation stage, except the caseins. Crude protein showed a lowering until 100-129 DIM, and then values are statistically not different until the end of lactation. The same trend was found for whey proteins, where the lowering was registered until 130-159 DIM. The casein to whey proteins ratio value ranged from 0.63 (100–129 DIM) to 0.80 (220–249 DIM). Proteose-peptones described a drop at the middle of lactation (110-189 DIM) and then increased until the end of the lactation. 

Ash as well as Ca, P, Mg, Na, Fe, and Zn contents were affected by the days in milk (Table 4), whereas no significant variations were reported for K, Cl, and Cu. Ash, Ca, P, and Mg showed an irregular downward trend, with a marked drop after 160 DIM (for ash, Ca, and P) and after 190 DIM (for Mg). A weak increase of ash, Ca, P, and Mg was observed in the last period of lactation. However, the values are always lower than those recorded in the first recorded period (40–69 DIM). The Na content showed a peak at the middle of lactation (130–189 DIM). In the first 160 DIM, no significant variations of the molar ratio between Ca and P was recorded and this value was always higher than 1. After that, a significant decrease was observed, which remains statistically constant until the end of lactation. With regard to the micro elements, the content of Zn was affected by the stage of lactation (*p* ≤ 0.01) with the highest values at 130–150 DIM. However, it did not show a clear trend, whereas Cu was steadily present in a low amount (<0.5 mg/kg).

Fe increased very significantly with a maximum of 4.69 mg/kg during the middle of lactation (160-189 DIM), which reached a value more than three times higher with respect to the early lactation.

In Table 5, the Pearson correlations between the milk production level, some milk parameters, and macro mineral content of Ragusano donkey milk samples are presented. 

Milk yield was positively correlated with dry matter and lactose content whereas it was negatively correlated with proteose-peptones and somatic cell count. Proteoso-peptones were positively correlated with the whey proteins content (r = 0.307, *p* ≤ 0.05, data not shown) as well as with the total bacterial count (r = 0.397, *p* ≤ 0.01, data not shown). Milk yield, somatic cell count, dry matter, and lactose content resulted in the parameters that showed a higher correlation with the minerals. The correlations were always positive with Ca, P, and Mg, and were always negative with K and with chloride. Moreover, a pH value showed a correlation, but with a low r value, with chloride and Na. Total ash was positively correlated with Ca and P content and with Mg content (moderately). The somatic cell count was positively correlated with Na and chloride, and negatively correlated with K. Crude protein and casein contents were positively correlated with Ca, P, and Mg, which are the macro elements that form the casein micelles. Moreover, casein positively correlated with K. Proteose-peptones were negatively correlated with milk yield and Na.

With regard to the correlations between minerals, Ca, P, and Mg were strongly and significantly correlated (Table 6). This is due to the fact that all three are involved in the construction of casein micelles. Na was positively correlated with Chloride, Fe, Cu, and Zn. A negative correlation was detected between Fe and Ca contents and a moderate correlation was detected between Na and Fe contents. Among micro-elements, Zn showed a major number of correlations with all other macro-elements and micro-elements. In particular, it is positively correlated with Ca, P, Mg, Na, and Cu, and negatively correlated with K. Only moderate and positive correlations were found between Cu and P, Ca, Na, and Fe milk content.

## 4. Discussion

The Ragusano breed, reared in a specialized dairy farm, confirmed its good aptitude to milk production, which was already reported [7,18]. Daily milk yield (1.64 ± 0.79 kg/day) was higher in comparison to other Italian breeds such as Amiata (735.0 ± 83.5 mL/milking) [9], Martina Franca (1469.2 ± 77.6 mL/day) [19], or Croatian Littoral Dinaric asses, which produce 172 ± 0.27 mL/milking [8] or Chinese donkeys with 1.28 ± 0.17 kg/day in 180 days of lactation [20]. Additionally, in this case, it must be specified that this milk is the milk produced after the removal of the foal from the mother. The observed differences can be attributed to the breed as well as to the breeding techniques, such as the number of milkings and the milking interval [21]. The milk produced by Ragusano donkeys confirmed the high level of lactose (on average 6.4 g/100 g) and the low protein content (on average 1.6 g/100 g) reported in all the studies on ass milk, which was carried out in different breeds and continents [22]. On the contrary, very low fat content observed, which was never higher than 0.32 g/100 g, seems to be a peculiar trait of Ragusano milk, as already reported [18], in contrast with the higher values (0.5–1.7 g/100 g) observed in Chinese [20], Greek (0.7–1.4 g/100g) [10], and other Italian donkey breeds (0.30–0.72%) [9,23]. The low-fat concentration provides a fresh, light, and clean taste to Ragusano’s ass milk and prevents the potential development of aftertaste. The rare sensory evaluations, which are carried out on donkey milk, reported this milk tasted as thin, with a slight sweet pleasant taste and with no persistent aftertaste [24], as well as an odor of grass and an almond’s flavor [21]. Overall, the low content of fat in Ragusano’s milk could make it particularly valuable for some categories of consumers, such as elderly. In asinine milk, the low-fat content as well as its quality in terms of proportion between saturated and unsaturated fatty acids, was reported useful for preventing and treating obesity [25] and atherosclerosis [26]. On the other hand, it must be taken into account that donkey milk contains too little fat to be treated as a full substitute for cow’s milk in human nutrition. For this reason, as attested by Tidona et al. [6], some paediatricians suggest the addition of safe oil to donkey milk [27] in order to reach a suitable energy intake for the infants. The protein fraction of Ragusano’s milk mainly consists of whey proteins. The low casein to whey proteins ratio, together with the high lactose content, makes donkey milk very similar to human milk. The casein content showed a wide variability at the individual level. This might be due to diverse expression levels of casein genes potentially linked to genetic polymorphisms, as reported in Ragusano donkeys by Criscione et al. [28]. In our samples, a relevant number of proteose-peptones (0.35 g per 100 g) was detected. The proteose-peptones, which have emulsifying and foaming properties, have not been investigated in donkey milk so far and no comparison with the literature is possible. With regard to the wide variability of the pH values, it seemed to reflect the large range of variability of the total microbial count and that of the somatic cells. In particular, the maximum values of these two parameters correspond to some rare milk samples that have been polluted or mastitic.

The minerals in milk play an important role in growth and skeletal structure development as well as in many other biological functions. In Ragusano’s milk, ash showed a very similar level to those reported in different breeds (0.3–0.4 g per 100 g) [22]. In addition, the percentage of ash on dry matter (4.40%) is in accordance with data reported by Salimei et al. [29]. Overall, the mineral content was lower than in the other milk samples except for the human one (0.2–0.3 g per 100 g) [30]. This evidence confirms that donkey milk is better than ruminant’s milk samples as a breast milk substitute for infants because the low ash content in this milk, together with a reduced amount of proteins, suits the limited renal capacity. Therefore, the renal load was reported to be very similar in both breast-fed infants and those fed donkey milk [31]. Furthermore, the low amount of minerals in donkey milk could be associated, reasonably, with a higher bioavailability, which occurs in human milk [31]. Among the macro elements, K was the most abundant, such as in human milk [32], goat milk, and cow milk [33,34], with an amount almost double the Ca content. This outcome is unexpected since, in the literature, Ca has always been reported to be the major element in the ass’s milk [10,29,35] as well as in mare milk [36]. The only exception is a research study by Bilandžić et al. [37] on seven Littoral-Dinaric donkeys investigated for nine months of lactation, which reported K as the main element. Another remarkable result is that P, which is generally reported linked to Ca and four times more abundant than Na [35], in our sample accounted for the same level of Na. The mean values of Magnesium (Mg) and Chloride were in the range previously observed in equidae milk (55.86 –112.94 mgL^−1^ and 150–198 ppm, respectively) [35,36], but different from Bilandžić et al. [37], which reported higher levels of Mg in Croatian donkey milk and horse milk (80.8 and 72.1 mg mgL^−1^, respectively). The Mg in Ragusano’s milk was much higher than in human milk (3–4 mg per 100 g) and about two to three times lower than in bovine (7–12 mg per 100 g and goat (10-36 mg per 100 g) milk [30]. The Cl^−^ level was higher than in mare milk (19 mg/100 g), but lower than in human (60–63 mg/100 g) and ruminant milk (57–209 mg per 100 g) [30]. The ratios between the main macro-minerals might be of great nutritional significance. Our study highlighted that a Ca to P ratio was in the range previously reported in other breeds (0.93–2.37 units) [38] and a Na to K ratio about three times higher than that (0.19) detected by Fantuz et al. [35]. Micro-elements (Fe, Zn, and Cu) resulted in the range reported by Salimei and Fantuz [38] in different breeds. Ragusano’s milk showed a higher level of Fe and Cu and the same amount of Zn than donkey milk collected in Italy and Croatia [17,37]. The high concentration of Fe in our samples of Ragusano’s milk is remarkable in comparison with the milk from other species [22,38]. Zn content was similar to that found in human milk but lower than in ruminant’s milk [30], whereas Cu values resulted in the range described in human [39], equine, bovine, and caprine milk [30].

### Effect of the Lactation Stage

The milk yield was significantly affected by the lactation stage in Ragusano jennies. The trend of the production, with a peak observed in the first recorded period (40–69 DIM), was in agreement with the previous reports in Italian [7,18,21,25,38], Chinese [20], and Croatian [8] breeds and populations. In Ragusano’s milk, the amount of total protein, as well as the casein and the other nitrogen fraction content, were higher at the beginning of the lactation. All the fractions’ content decreased constantly as reported in literature [7,18,20,38], except for casein, which was not significantly affected by the lactation stage. This last outcome is in contrast with Martini et al. [9]. The mineral composition is significantly affected by the lactation stage: a small but significant reduction was recorded between the 160–250 days of lactation. During the lactation, a significant reduction of ash content was reported in a Chinese donkey [20] from 60 to 180 days from parturition. The major minerals (Ca, P, and Mg) decreased significantly from the early to the middle lactation in our samples, as previously found in 16 Martina Franca asses (from 46 to 142 days of lactation) [35] and in mare [36]. The other macro minerals (K and Na) did not show a clear trend, which is in agreement with Fantuz et al. [35]. Our results are in contrast with those from Fantuz et al. [17], which reported that Cu concentration clearly decreased during the lactation whereas Zn and Fe showed inconsistent trends. Furthermore, the results conflict with the decreasing trend for the Ca to P ratio and a rather constant value of the Na to K ratio recorded in the first 150 days of lactation by Fantuz et al. [35]. 

The observed correlations between milk yield and lactose content, somatic cells count, and proteose-peptones content can be explained by the role of osmotic regulation that the lactose plays in the mammary glands. In other species (i.e., bovine), a strict relation between lactose content and the milk yield level is well known. An increased somatic cell count in milk is commonly associated with an inflammation process of the mammary gland. The inflammatory response is usually characterized by an intensification of the enzymatic proteolytic activity, responsible for the increase of the proteose-peptone content in milk, and by a decrease of the secretory activity, which results in reduced milk production [40]. Moreover, the milk secreted is characterized by some alterations of the chemical composition such as by a decrease of Ca, K, P, and Mg and an increase of Na and chloride content [40].

The strong correlation between Na, chloride, and Fe is imputable to the strong correlation that exist between the somatic cell, Na, and chloride. In fact, in the presence of mastitis, there is a passage of some blood components in the milk, with an increase of NaCl and Fe content. This observation was confirmed by the negative correlation between Fe and Ca content and the moderate correlation between Na and Fe content. 

The correlations of crude protein as well as of casein content and of major elements (Ca, P, and Mg) can be explained by the association of these minerals into the casein micelle as inorganic calcium phosphate (or as magnesium phosphate) or as organic phosphorus directly linked to casein amino acids, as previously described by Malacarne et al. [41].

Our results differ from Bilandžić et al. [37], who found a strong and positive association between Ca, Na, and Cu, and a negative correlation of K with Ca, Na, and Cu. These different findings could be attributed to the breed factor but also to the different breeding system and productive level. Lastly, results of the present research, for the positive correlations of Zn with Mg and Zn with Cu, are in agreement with the study of Fantuz et al. [42]. 

In light of the results shown here, the nutritional quality and the mineral composition of Ragusano donkey milk over the lactation are only partially in agreement with those reported in other donkey breeds. These discrepancies could be due to differences in breed, the breeding system, and lactation length. With regard to the diet effects on mineral composition, some studies found that the mineral supplementation in the diet had no effect on major and minor elements in donkey milk [17,35], while another study reported that the levels of some minor elements (such as Fe, Zn, and Cu) can be affected by the diet and the geographical origin [43]. Furthermore, from a nutritional point of view, it is worth noting that the total amount of minerals is of minor importance compared to their bioavailability and breast milk is a clear example. Given that no mineral deficiency has been reported in infants who consumed ass’s milk, it is reasonable to assume that the minerals in this milk have a good bioavailability, which is similar to human milk. Mineral bioavailability is subject to the binding of ligand molecules, but it also depends on the whole composition of milk. In particular, it was supposed that casein may act as limiting factor for the absorption of some minor elements, especially in infants with a limited digestive function [44]. Although limited data are available regarding compounds that bind minerals in donkey milk, it is possible that the low casein to whey protein ratio may confer a higher mineral bioavailability for donkeys. In clinical studies, despite the general small amount of total minerals, the consumption of ass’s milk was associated with a good intestinal absorption of calcium, which is likely enhanced by the high level of lactose. This is essential for the bone’s mineralization in infants and the elderly [31]. Similarly, it is reported that, despite the low content, Zn utilization was higher from human milk, than from the bovine counterpart because of the lower casein content of breast milk [39].

## 5. Conclusions

Donkey milk is increasingly being proposed as a natural alternative milk for various categories of consumers, especially infants and the elderly population, but its potential production, gross, and mineral composition have not been deeply investigated yet. This milk is not standardized. High variability is reported in the limited literature. Results are often contradictory, likely due to both genetic and environmental factors. Nevertheless, the Ragusano breed is considered the main Italian dairy donkey breed. In conclusion, the Ragusano breed showed a good aptitude to milk production and the composition of the milk confirmed its nutritional quality. However, in light of the results presented here, the gross and the mineral composition of Ragusano milk, investigated over nine months of lactation, are only partially in agreement with those reported for other breeds. As a whole, the numerous inconsistencies currently recorded suggest that the studies conducted until now have failed to outline a standardized nutritional profile of donkey milk. In particular, the detailed mineral fraction needs to be further investigated, in order to provide more conclusive insights about the nutritional properties of donkey milk and its potential derivatives.

## Figures and Tables

**Table 1:** Descriptive statistics of chemical composition parameters, physico-chemical properties, total bacterial cell counts, and somatic cell counts of individual Ragusano donkey milk samples (62 whole milk samples collected from nine donkeys).

Parameters		Means	SD ^1^	Minimum	Maximum
Milk yield ^2^	kg/day	1.64	0.79	0.38	3.30
Dry matter	g/100 g	8.19	0.60	6.63	9.55
Lactose	g/100 g	6.07	0.44	4.90	6.72
Fat	g/100 g	0.16	0.06	0.07	0.32
Crude protein	g/100 g	1.34	0.20	0.93	1.88
Whey protein	g/100 g	0.78	0.09	0.60	1.02
Casein	g/100 g	0.56	0.16	0.14	1.14
Casein number	%	41.07	7.34	13.85	60.52
NPN × 6.38	g/100 g	0.17	0.05	0.07	0.26
True protein	g/100 g	1.17	0.18	0.75	1.70
Proteose-peptones	g/100 g	0.35	0.07	0.20	0.57
Ash	g/100 g	0.36	0.06	0.25	0.48
pH	Value	7.35	0.14	7.09	7.73
Total bacterial count	FCU ^3^/mL	20,097	30,740	2000	103,000
Somatic cell count	Cells/mL	103,935	68,631	9000	255,000

^1^ SD = Standard deviation. ^2^ Milk yield production from 6 a.m. to 3 p.m. ^3^ FCU = Forming colony units.

**Table 2:** Descriptive statistics of macro mineral and micro mineral content of individual Ragusano donkey milk samples (62 whole milk samples collected from nine donkeys).

Parameters		Means	SD ^1^	Minimum	Maximum
Ca	mg/100 g	54.36	21.10	20.76	92.43
P	mg/100 g	43.44	9.50	20.04	73.89
Mg	mg/100 g	6.13	1.05	4.08	8.15
Na	mg/100 g	43.77	14.64	21.68	99.36
K	mg/100 g	110.27	17.41	62.65	146.35
Chloride	mg/100 g	36.57	14.43	20.53	81.70
Zn	mg/kg	2.24	0.70	0.99	4.63
Fe	mg/kg	2.29	1.72	0.57	9.59
Cu	mg/kg	0.31	0.18	0.15	1.17
Ca to P ratio	mol/mol	0.95	0.25	0.30	1.46
Na to K ratio	mol/mol	0.70	0.27	0.28	1.44

^1^ SD = Standard deviation.

**Table 3:** Variation of chemical composition parameters and physico-chemical properties of Ragusano donkey milk during lactation (62 whole milk samples). Least square mean values ± standard errors.

Parameters		Day of Milking																
		40–69 Days (*n* ^1^ = 9)		70–99 Days (*n* = 9)		100–129 Days (*n* = 9)		130–159 Days (*n* = 9)		160–189 Days (*n* = 9)		190–219 Days (*n* =7)		220–249 Days (*n* = 5)		250–279 Days (*n* = 5)		*p* ^4^
		LSM ^2^	SE ^3^	LSM	SE	LSM	SE	LSM	SE	LSM	SE	LSM	SE	LSM	SE	LSM	SE	
Milk yield	kg/day	2.11	0.12 d	1.91	0.12 c,d	1.98	0.12 c,d	2.06	0.12 c,d	1.73	0.12 c	0.81	0.14 b	0.49	0.17 a,b	0.13	0.17 a	***
Dry matter	g/100 g	8.60	0.15 d	8.45	0.15 c,d	8.63	0.15 d	8.07	0.15 b,c	7.52	0.15 a	7.79	0.17 a,b	8.28	0.21b c,d	7.7	0.21 a,b	***
Lactose	g/100 g	6.30	0.10 c	6.28	0.10 c	6.23	0.10 c	6.08	0.10 b,c	5.71	0.10 a	5.80	0.11 a,b	5.84	0.13 a,b	5.7	0.13 a	***
Fat	g/100 g	0.12	0.01 a	0.11	0.01 a	0.19	0.01 c	0.18	0.01 b,c	0.19	0.01 c	0.21	0.02 c	0.14	0.02 a,b	0.09	0.02 a	***
Crude protein	g/100 g	1.59	0.05 c	1.42	0.05 b	1.34	0.05 a,b	1.26	0.05 a	1.28	0.05 a	1.28	0.06 a,b	1.27	0.07 a,b	1.28	0.07 a,b	**
Whey protein	g/100 g	0.90	0.02 d	0.83	0.02 c	0.82	0.02 b,c	0.75	0.02 a	0.73	0.02 a	0.72	0.02 a	0.71	0.02 a	0.76	0.02 a,b	***
Casein	g/100 g	0.69	0.05	0.60	0.05	0.52	0.05	0.51	0.05	0.55	0.05	0.56	0.06	0.57	0.07	0.52	0.07	NS
Casein number	%	43.45	2.43	41.78	2.43	38.7	2.43	39.93	2.43	42.48	2.43	41.66	2.81	43.56	3.38	38.07	3.38	NS
NPNx6.38	g/100 g	0.22	0.01 d	0.17	0.01 b,c	0.19	0.01 c,d	0.18	0.01 b, c,d	0.15	0.01 a,b,c	0.12	0.02 a	0.14	0.02 a,b	0.14	0.02 a,b	**
True protein	g/100 g	1.37	0.05 c	1.25	0.05 b,c	1.15	0.05 a,b	1.08	0.05 a	1.12	0.05 a,b	1.16	0.06 a,b	1.13	0.07 a,b	1.14	0.07 a,b	*
Proteoso peptone	g/100 g	0.39	0.02 c,d	0.36	0.02 b,c,d	0.31	0.02 a,b	0.30	0.02 a	0.31	0.02 a	0.35	0.02 a,b,c	0.37	0.02 b,c,d	0.43	0.02 d	**
pH	Value	7.28	0.03 a	7.46	0.03 c	7.32	0.03 a,b	7.39	0.03 b,c	7.41	0.03 c	7.38	0.04 a,b,c	7.29	0.04 a,b	7.27	0.04 a	**
Total Bacterial	FCU/mL	3.84	0.12 a,b	4.73	0.12 c	3.70	0.12 a	3.84	0.12 a,b	3.81	0.12 a,b	3.80	0.14 a b	3.87	0.17 a,b	4.23	0.17 b	***
Somatic cell	Cells/mL	5.18	0.10c	4.82	0.10 a,b	4.97	0.10 b,c	5.09	0.10 b,c	4.85	0.10 a,b	4.63	0.12 a	4.71	0.15 a,b	4.78	0.15 a,b	*

^1^*n* = number of samples. ^2^ LSM = least square mean values. ^3^ SE = Standard errors. ^4^ *p* = significance of difference: a. b. c. d. different for *p* ≤ 0.05. NS. *p* > 0.05. * *p* ≤ 0.05. ** *p* ≤ 0.01. *** *p* ≤ 0.001.

**Table 4:** Variation of ash, macro mineral content, and micro mineral content of Ragusano donkey milk during lactation (62 whole milk samples). Least square mean values ± standard errors. Measured units for each parameter are the same as in Table 1 and Table 2.

Parameters	Day of Milking																
	40–69 Days (*n* ^1^ = 9)		70–99 Days (*n* = 9)		100–129 Days (*n* = 9)		130–159 Days (*n* = 9)		160–189 Days (*n* = 9)		190–219 Days (*n* = 7)		220–249 Days (*n* = 5)		250–279 Days (*n* = 5)		*p* ^4^
	LSM ^2^	SE ^3^	LSM	SE	LSM	SE	LSM	SE	LSM	SE	LSM ^2^	SE	LSM	SE	LSM	SE	
Ash	0.41	0.01 d	0.38	0.01 c,d	0.40	0.01 c,d	0.38	0.01 c	0.32	0.01 a	0.31	0.01 a	0.33	0.01 a,b	0.36	0.01 b,c	***
Ca	78.85	3.08 d	64.68	3.08 c	69.51	3.08 c	61.02	3.08 c	37.97	3.08 a,b	30.89	3.57 a	35.96	4.29 a,b	46.50	4.29 b	***
P	52.56	2.45 c	46.36	2.45 b,c	48.56	2.45 b,c	46.31	2.45 b,c	38.49	2.45 a	33.35	2.84 a	33.16	3.42 a	41.16	3.42 a,b	***
Mg	7.21	0.19 d	6.51	0.19 c	6.60	0.19 c	6.34	0.19 c	5.66	0.19 b	5.02	0.22 a	5.07	0.26 a,b	5.63	0.26 a,b	***
Na	40.69	4.18 a	35.01	4.18 a	42.59	4.18 a b	54.79	4.18 b	53.32	4.18 b	48.40	4.85 a,b	33.74	5.83 a	37.93	5.83 a	*
K	108.14	5.17	104.97	5.17	109.37	5.17	97.38	5.17	115.98	5.17	122.69	5.99	114.16	7.21	123.37	7.21	NS
Cl^–^	31.42	3.71	31.36	3.71	32.54	3.71	36.06	3.71	43.25	3.71	45.10	4.30	44.86	5.17	43.11	5.17	NS
Zn	2.35	0.19 b	2.02	0.19 a,b	2.31	0.19 b	3.02	0.19 c	2.17	0.19 b	1.52	0.23 a	1.90	0.27 a,b	2.55	0.27 b,c	**
Fe	1.41	0.42 a	1.21	0.42 a	1.20	0.42 a	3.29	0.42 b	4.69	0.42 c	2.87	0.49 b	1.77	0.59 a,b	1.83	0.59 a,b	***
Cu	0.33	0.05	0.22	0.05	0.30	0.05	0.40	0.05	0.33	0.05	0.22	0.06	0.22	0.07	0.48	0.07	NS
Ca:P	1.16	0.05 c	1.08	0.05 c	1.10	0.05 c	1.01	0.05 b,c	0.84	0.05 a	0.69	0.06 a	0.79	0.07 a	0.86	0.07 a,b	***
Na:K	0.64	0.08 a	0.57	0.08 a	0.72	0.08 a	1.01	0.08 b	0.78	0.08 a,b	0.66	0.10 a	0.50	0.12 a	0.51	0.12 a	*

^1^*n* = number of samples. ^2^ LSM = least square mean values. ^3^ SE = Standard errors. ^4^ *p* = significance of difference: a. b. c. d different for *p* ≤ 0.05. NS. *p* > 0.05. * *p* ≤ 0.05. ** *p* ≤ 0.01. *** *p* ≤ 0.001.

**Table 5:** Pearson correlations between the milk production level and some milk parameters, physico-chemical properties, somatic cell counts, and macro mineral and micro mineral content of Ragusano donkey milk samples.

Parameters	Milk Yield	P	Ca	Mg	Na	K	Chloride
Milk yield	-	0.418 ***	0.394 **	0.308 *	0.091	−0.321 *	−0.279 *
Dry matter	0.348 **	0.308 *	0.456 ***	0.376 **	−0.373 **	−0.218 *	−0.370 **
Lactose	0.504 ***	0.223 *	0.294 **	0.256 *	−0.403 **	−0.416 **	−0.613 ***
Crude protein	0.139	0.371 **	0.521 ***	0.553 ***	0.096	0.172	0.174
Casein	0.072	0.180 *	0.285 *	0.355 **	0.150	0.258 *	0.225
Proteoso-peptones	−0.337 **	−0.013	0.086	0.125	−0.271 *	−0.110	−0.128
Ash	0.248	0.751 ***	0.888 ***	0.550 ***	0.121	−0.020	0.069
pH	0.14	−0.017	0.120	−0.192	0.283 *	0.098	0.339 **
Somatic cell	−0.339 **	0.374 **	0.447 **	0.141	0.276 *	−0.199 *	0.123 *

Significance of correlation: * *p* ≤ 0.05, ** *p* ≤ 0.01, *** *p* ≤ 0.001.

**Table 6:** Pearson correlations between macro mineral and micro mineral content of Ragusano donkey milk samples.

Parameters	P	Ca	Mg	Na	K	Cl	Fe	Cu	Zn
P	-	0.743 **	0.547 **	0.288 *	−0.175	−0.029	−0.120	0.269 *	0.482 **
Ca		-	0.665 **	0.026	−0.225	−0.170	−0.309 *	0.267 *	0.500 **
Mg			-	−0.015	−0.093	−0.268 *	−0.147	0.236	0.343 **
Na				-	0.019	0.574 **	0.505 **	0.308 *	0.341 **
K					-	0.419 **	0.025	−0.172	−0.384 **
Cl						-	0.178	−0.027	−0.014
Fe							-	0.329 **	0.197
Cu								-	0.504 **
Zn									-

Significance of correlation: * *p* ≤ 0.05, ** *p* ≤ 0.01, and *** *p* ≤ 0.001.
